# Dietary intake and meal patterns among young adults with high caries activity: a cross-sectional study

**DOI:** 10.1186/s12903-022-02227-w

**Published:** 2022-05-19

**Authors:** Annie Guo, Ulla Wide, Louise Arvidsson, Gabriele Eiben, Magnus Hakeberg

**Affiliations:** 1 Department of Pediatrics, Institute of Clinical Sciences, Sahlgrenska Academy, Gothenburg, Sweden; 2 Department of Behavioral and Community Dentistry, Institute of Odontology, Sahlgrenska Academy, University of Gothenburg, P.O. Box 450, 40530 Gothenburg, Sweden; 3 Public Dental Service, Region Västra Götaland, Sweden; 4 Department of Public Health, School of Health Sciences, University of Skövde, Skövde, Sweden

**Keywords:** Oral health, Dental caries, Sugar, Dietary intake, Meal patterns, Young adults

## Abstract

**Background:**

Dental caries is a multifactorial disease that is highly dependent on diet, where a lower consumption and intake frequency of sugar would be favorable. The aims were (i) to examine dietary intake and meal patterns, more specifically sugar intake and foods high in sugar, among young adults with high caries activity, and (ii) to investigate the association between dietary and meal patterns consumption, and level of caries activity.

**Methods:**

This study presents baseline data from an ongoing randomized controlled trial. A total of 50 young adults (aged 23.0 ± 3.0 years) with ≥ 2 decayed tooth surfaces were included. Dietary intake was captured with a 59-item food frequency questionnaire (FFQ) and a three-day food diary. Adherence to dietary guidelines was analyzed by comparing the dietary intake to the Nordic Nutritional Recommendations (NNR) 2012 and by using the Healthy Dietary Adherence score (HDAS). Participants were categorized into two groups: (i) the Caries group with 2–4 decayed surfaces, and (ii) the High caries group with ≥ 5 decayed surfaces.

**Results:**

The High caries group reported a statistically significantly higher snack and total meal intake compared to the Caries group, as well as a sugar intake exceeding the Nordic nutritional recommendations. The majority of the participants reported a high intake frequency (> 2.5/day) of sweet foods and drinks and less than one intake of fruit and vegetables, respectively, per day. Similar results were found when analyzing adherence by using the HDAS, where the lowest adherence according to dietary guidelines was shown for the food groups of sugar, whole meal products, and fruit and vegetables.

**Conclusion:**

The results indicated a high intake of sugar and low intake of fruit, vegetables, and fiber in high caries-active individuals.

## Background

Dental caries is a major health issue and has been estimated to affect close to 2.3 billion adults and more than 530 million children worldwide [1, 2]. The disease is associated with negative consequences for the individual’s quality of life as advanced states of dental caries cause loss of tooth substance and conditions and symptoms such as pain, infection, abscesses and sepsis [3]. Moreover, dental caries has a great impact on health care [2], and has been estimated to account for 5–10% of the total health care budgets in industrialized countries [3].

Dental caries is a multifactorial disease highly dependent on behavioral factors, where oral hygiene, fluoride use and dietary habits are of great importance [2]. Excessive intake of fermentable carbohydrates has been reported as one of the main drivers, as it affects the integrity of the teeth; plaque and biofilm pH [4, 5]. In addition to the amount of carbohydrates, the intake frequency of carbohydrates has also been suggested to be an important key factor for the risk of developing dental caries [6]. Recent research has found positive associations between the frequency of sugar consumption and caries in both adults and children [7, 8]. Hence, the frequency and amount of sugar consumption are of great importance in the prevention of dental caries [9].

To reduce the risk of non-communicable diseases in adults and children, particularly unhealthy weight gain and dental caries, the World Health Organization (WHO) has recommended limiting the intake of free sugars for adults and children to less than 10% of the energy intake (E%) [2]. To further reduce the risk of dental caries, a reduction to less than 5 E% have been suggested. Today, sugar consumption is increasing, and the dietary guidelines regarding sugar intake are exceeded by many populations worldwide [10]. A recent review assessing a European population found that the relative intake of sugar ranges between 15 and 21 E% in adults and between 16 and 26 E% in children [11]. Similar findings have been shown in other countries such as the US and Brazil [12].

A dietary shift towards less consumption of sugar would not only benefit oral health [2, 13, 14]. Previous research has suggested a common risk factor approach where several chronic diseases might be prevented by targeting the common risk factors, as the favorable effects would target not only one single disease but several conditions simultaneously [14]. For instance, food high in sugars has been strongly associated with adverse health effects and chronic diseases such as obesity, cardiovascular disease and various types of cancer [2, 13]. In addition, a high intake of fruit, vegetables and whole grains has been found to be protective [13]. It has been suggested that dietary consultation in dental care should be consistent with national dietary guidelines and the advice given by other health professionals [6].

Food preferences and meal patterns are formed during early childhood, up to the beginning of adulthood [15, 16]. Thus, healthy eating at a young age is decisive for the eating habits the individual brings into adult life. In Sweden, women and men aged 18–30 years have reported the least healthy eating habits [13]. To the authors’ knowledge, dietary intake and meal patterns, specifically among young adults with high caries activity, have not yet been examined.

Therefore, the aims of the present study were (i) to examine dietary intake and meal patterns, more specifically sugar intake and foods high in sugar, among young adults with high caries activity, and compare them with the general population and with healthy dietary guidelines, (ii) to investigate the association between dietary intake and meal patterns, and level of caries activity.

## Methods

### Study design and study sample

This study used baseline data measurements from the ongoing Dietary and Oral Health study, a randomized controlled trial evaluating a manual based dietary consultation provided by a licensed dietician, in general dental care. Participants were recruited between April 2019 and May 2021 at a Public Dental Service Clinic in Region Västra Götaland, Sweden. The study was approved by the Regional Ethical Review Board in Gothenburg (reg. no. 185-16; reg. no. T860-18) and was conducted in accordance with the Helsinki Declaration. The inclusion criteria were 18–30 years of age and a minimum of two registered manifest approximal caries lesions (dentin caries). The exclusion criteria were a psychiatric or neuropsychiatric diagnosis and inability to understand written information in Swedish. Eligible persons were asked to participate in the study during their ordinary dental examination. Informed consent was obtained from all participants. The sample size calculation for the RCT included as primary outcome the variables snacks and sweet drinks. Using a mean difference of 1.0, an estimated SD of 1.2, significance level of 0.05 a sample size of n = 24 per group was calculated. An expected drop-out made us include a group size of n = 32. However, for this paper, the analysis of baseline data included a total n = 50 since data for food measures were missing for n = 14.

### Anthropometrical and clinical measurements

Body mass index (BMI) was calculated as kg/m^2^ using height and weight according to the WHO definition [17]. Recording of the number and types of caries lesions was performed according to the standard applied in Region Västra Götaland (D1–D3, secondary caries), including recording of caries on four surfaces per tooth and proximal caries measured from radiological examinations. Self-reported oral health was measured with a global question with four response options (poor, fair, good, or very good). Oral hygiene was assessed with questions regarding tooth-brushing, dental flossing, and additional fluoride (besides toothpaste) with six response alternatives (three times a day or more; twice a day; once a day; several times a week; once a week; more seldom/never). Dental care attendance was captured with the question ‘How often do you visit a dental care clinic?’, with five response alternatives (twice a year; once a year; once every other year; less than once every other year, only in case of acute discomfort; never). Smoking status was reported as current smoking (yes or no). Sociodemographic characteristics were measured with questions regarding ethnicity (Swedish-born, other Nordic countries, other countries), employment (employed including sick leave, parental leave and labor market measures, unemployed, student), and mother and father’s education (elementary school, high school, university). The participants were asked to report their average physical activity during a week by choosing either inactive (mostly sedentary during working hours, exercise a maximum of once/week), moderately active (mostly sedentary during working hours, exercise 2–3 times/week) or very active (physically demanding work and exercise > 4 times/week). The study sample was divided into two groups depending on the number of decayed tooth surfaces per individual (manifest, dentine (D3), and secondary caries). A ‘Caries group’ (n = 25) (2–4 approximal caries surfaces) and a ‘High caries group’ (n = 25) (≥ 5 approximal caries surfaces) were set up using the median value, for analysis purposes.

### Dietary intake

Dietary intake was assessed by using a three-day self-reported food diary. Participants were asked to record their usual food consumption during two weekdays and one day of the weekend. Energy and nutrient content were estimated by using the software DietistNet (version 20.05.22) and data from food manufacturers. Standard Swedish recipes [18] were used to estimate the nutritional value when the type of food was missing in DietistNet. The reported dietary intake was compared with the Nordic Nutritional recommendations (NNR) 2012 [13], which emphasize a dietary composition with 25–40 E% total fat, ≤ 10 E% saturated fat (SFA), 10–20 E% monosaturated fat (MUFA), 5–10 E% polysaturated fat (PUFA), 46–60 E% carbohydrates, < 10 E% added sugar, 10–20 E% protein and ≤ 5 E% alcohol. Due to limited data on added sugar in DietistNet, sugar was defined as sucrose only. Intake occasions were defined as intake of energy-containing foods, including beverages, and categorized into main meals (composite, prepared dishes, or dishes reported as main meals), snacks (food/beverages between main meals), and total meals (sum of main meals and snacks per day). Food groups were categorized into sweet drinks, light drinks, vegetables, fruits, and snacks. The variables analyzed were energy (kcal), fiber (g/d), macronutrients, fatty acids (E%), sucrose, alcohol, and intake occasions as frequencies (n). As in previous research [19], participants reporting a daily energy intake of less than 800 kcal or over 3500 kcal were considered as possible under-reporters or over-reporters.

### Meal patterns

The participants were asked to report their usual meal patterns during the preceding month in a 59-item Food Frequency Questionnaire (FFQ), a questionnaire previously used in the IDEFICS study and developed in the I.Family study [20]. The following food groups were asked for: vegetables, fresh fruit, drinks, cereals, milk, yoghurt, fish, meat and meat products, eggs and mayonnaise, soy products and meat substitutes, cheese, spreadable toppings (e.g., butter, jam, chocolate spread), cooking oil, grains, snacks, and alcohol. The response options in the FFQ were: never/less than once a week, 1–3 times/week, 4–6 times/week, once/day, twice/day, 3 times/day and ≥ 4 times/day.

### The healthy dietary adherence score

Adherence to a healthy diet was captured by calculating the Healthy Dietary Adherence Score (HDAS) from the FFQ. The HDAS complies with European dietary guidelines regarding a recommended intake of 400-500 g fruits and vegetables per day, fish 2–3 times per week, choice of whole meal products (e.g., whole meal bread, dark roll, dark crispbread) when possible, reduced intake of fat, more specifically saturated fats, and limited intake of refined sugars [21]. The total HDAS score and adherence to its components were analyzed by using the group median as a cutoff (median included in the lower group) to define higher and lower adherence. A more detailed description of the HDAS can be found elsewhere [21, 22].

### The Swedish national dietary survey

The Swedish national dietary survey (Riksmaten adolescents 2016–17) [23] was used to compare the reported dietary intake with the general Swedish population. This study included a total of 3477 students. To enable comparison, only boys and girls in grade 11, aged 17–21 (n = 1000) were included in the analysis.

### Statistical analyses

The descriptive statistics used were means, medians, standard deviations (SD) and frequencies. The statistical methods applied were the independent T tests for normally distributed variables and the Mann–Whitney U test for non-normally distributed variables. Categorical variables were analyzed using Pearson’s chi-squared test. The significance level was set to *p* < 0.05. Bonferroni corrections for multiple comparisons were applied to the sociodemographic and clinical characteristics and dietary intake, giving significance levels of *p* < 0.004 (Table 1) and *p* < 0.003 (Table 2), respectively. Analyses were executed in the SPSS software (version 27).

**Table 1:** Sociodemographic and clinical characteristics of all participants (*n* = 50), categorized into the Caries group (2–4 approximal caries surfaces) and High caries group (≥ 5 approximal caries surfaces)

	Total group (*n* = 50)	Caries group (*n* = 25)	High caries group (*n* = 25)	p value
Age, years, mean (SD)	23.1 (3.0)	22.5 (3.1)	23.6 (2.8)	0.205
Body mass index, kg/m^2^, Mean (SD)	26.4 (5.6)	26.7 (6.0)	26.1 (5.3)	0.635
Body mass index, %				0.309
Underweight	4	4	4	
Normal weight	38	32	44	
Overweight	36	48	24	
Obese	22	16	28	
Caries, Mean (SD) Median	5.1 (3.5) 4.5	2.8 (0.7) 3.0	7.3 (3.7) 6.0	< 0.001
Self-rated oral health, %*				0.394
Poor	14	8	20	
Fair	38	36	40	
Good	44	52	36	
Very good	2	4	0	
Dental care attendance, % often*	84	96	75	0.165
Sex, % female	40	32	48	0.387
Smoking status, % smoking	20	20	20	1.000
Ethnicity, % Swedish-born^#^*	40	48	33	0.296
Employment, %				0.702
Student	40	44	36	
Employed	44	44	44	
Unemployed	16	12	20	
Mother’s education, %*				0.405
Elementary school	34	32	36	
High school	48	44	52	
University	16	24	8	
Father’s education, %*				0.538
Elementary school	52	44	60	
High school	36	44	28	
University	10	12	8	
Physical activity, %				0.519
Inactive	30	24	36	
Moderately active	26	24	28	
Very active	44	52	46	

BMI, body mass index; n, number; SD, standard deviation

^*^n = 49

^#^Including Nordic-born n = 1

**Table 2:** Reported mean (SD) intake of nutrient content, diet composition, intake occasions and food frequency in all participants and categorized into a Caries group (2–4 approximal caries surfaces) and a High caries group (≥ 5 approximal caries surfaces), compared with the recommended daily intake, data for adults in the Nordic Nutritional Recommendations 2012 (NNR) and Riksmaten Adolescents 2016–17

	NNR range	Riksmaten (n = 1000) mean (SD)	Total group (n = 50) mean (SD)	p value	Caries group (*n* = 25) mean (SD)	High caries group (*n* = 25) mean (SD)	p value
Energy, kcal	–	2 293 (573)	1854 (376)	< 0.001	1832 (273)	1875 (462)	0.639
Fiber, g	25–35	20 (7)	16 (5)	< 0.001	15 (5)	16 (5)	0.587
Diet composition, E%							
Total fat	25–40	36 (4)	36 (7)	0.5199	36 (7)	35 (8)	0.759
Protein	10–20	17 (4)	17 (5)	0.5199	17 (5)	16 (5)	0.455
Carbohydrates	45–60	45 (6)	47 (9)	0.9505	46 (9)	48 (10)	0.562
SFA	< 10	14 (2)	14 (3)	0.5199	14 (3)	13 (3)	0.350
MUFA	10–20	14 (2)	13 (5)	0.9265	13 (4)	14 (5)	0.524
PUFA	5–10	5 (1)	4 (1)	< 0.001	4 (1)	4 (1)	0.389
Sucrose	< 10	9 (3)	9 (6)	0.5199	8 (4)	10 (7)	0.467
Alcohol	< 5	–	0 (1)		0 (1)	0 (1)	0.986
Intake occasions, *n*							
Main meals	–	–	2.4 (0.5)		2.5 (0.4)	2.4 (0.7)	0.570
Snacks	–	–	2.5 (1.5)		2.0 (0.9)	3.2 (1.8)	0.003^a^
Total meal intake	–	–	5.0 (1.5)		4.5 (1.0)	5.6 (1.8)	0.033^a^
Food frequency, *n*							
Sweet drinks	–	–	1.6 (1.1)		1.5 (1.1)	1.7 (1.1)	0.286
Light drinks	–	–	0.5 (0.6)		0.5 (0.7)	0.5 (0.5)	0.349
Vegetables	–	–	0.8 (0.6)		0.7 (0.6)	0.8 (0.7)	0.732
Fruits	–	–	0.6 (0.6)		0.4 (0.5)	0.8 (0.6)	0.019^a^
Sweets and snacks	–	–	1.1 (1.4)		0.9 (0.9)	1.3 (1.7)	0.678

SD, standard deviation; E%, energy percentage; SFA, saturated fat; MUFA, monosaturated fat; PUFA, polysaturated fat

^a^Ns after Bonferroni correction

## Results

### Participant characteristics

**Fig. 1:**
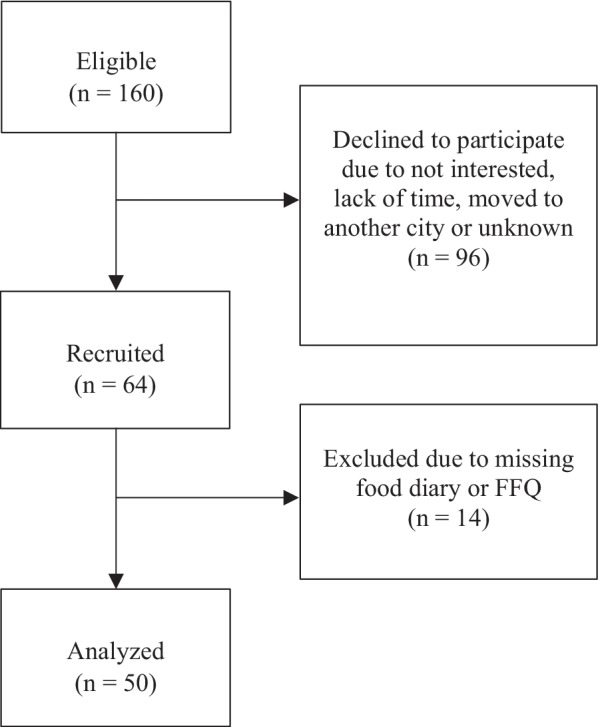
Flow diagram of participant enrolment

A total of 64 participants were recruited to this cross-sectional study, 50 of whom completed the dietary records (Fig. 1). Baseline characteristics are shown in Table 1. The participants had a mean age of 23 years and a mean number of 5.1 caries surfaces. The mean BMI was 26.4, which means that most of the participants were classified as either overweight or obese (36% and 22%, respectively). Approximately 50% of all participants rated their oral health to be poor or fair. Most of the study group members were males and a fifth of all participants reported being smokers. Regarding oral hygiene, around 75% reported brushing their teeth and using toothpaste twice a day or more, 47% reported using dental floss once a day or more and 51% reported using additional fluoride once a day.

### Dietary intake and meal patterns

Table 2 presents the nutrient content, diet composition, intake occasions and food frequency reported in the food diaries. The study group reported a mean intake of 1854 kcal/day and a fiber intake below the recommended intake. Compared with Riksmaten adolescents 2016–2017, the study group reported a lower energy and fiber intake (p < 0.001). Specifically, the study group complied with the recommended intake of total fat, protein, and carbohydrates and had a low consumption of alcohol. However, the reported intake of SFA and PUFA did not comply with the recommendations, with the intake of SFA being estimated as too high.

In the total study group, the reported mean intake of sugar was within the dietary recommendations, although excessive in the High caries group. The study group also tended to have a higher intake of sugar during the weekend than on weekdays (p = 0.003) and exceeded the recommended intake of < 10 E% sugar during the weekend (data not shown). Similar results were found when comparing weekdays and weekends in the High caries group. None of the caries groups had a reported intake within the recommended intake of < 5 E% sugar for reduced risk of caries.

Reported intake occasions in the study group showed that the High caries group had a higher mean intake of snacks between main meals (p = 0.003) and a higher total meal intake (p = 0.033) compared to the Caries group (Table 2). The total study group reported a mean intake frequency of 1.6 sweet drinks and 1.1 sweets and snacks, which generated a total daily intake of 2.7 intake occasions of sweet food and beverages. Comparing weekdays and weekends regarding food frequencies, the study group had a higher intake frequency of sweets and snacks (p = 0.011). All participants reported an intake of vegetables and fruit of less than once a day, with a tendency towards a higher intake of fruit in the High caries group than in the Caries group (p = 0.019).

### Healthy dietary adherence score

In the study group, a total of 52% reported low adherence to the HDAS. Comparison between the individual food groups showed lower adherence to the healthy dietary recommendations regarding sugar, whole meal, fruit, and vegetables. A higher adherence to the recommendations regarding fat and fish were shown. There were no significant differences in adherence to the individual food scores between the two caries groups.

## Discussion

This cross-sectional analysis of young adults with severe caries disease with respect to dietary intake and meal patterns indicated a high intake of sugar and a low intake of fruit, vegetables, and fibers. Few epidemiological studies have measured both the intake frequency and the amount of sugar with regard to dental caries [24]. To our knowledge, this is the first cross-sectional study evaluating dietary intake and meal patterns among young adults with high caries activity. Previously, associations between meal patterns, sugar intake and caries have mainly been examined separately and in younger populations. The present study shows that individuals with high caries activity had a high snack intake and exceeded the recommended intake of sugar. A recent systematic review, published in 2020, found that high-frequency consumption of processed foods containing sugar and starch was associated with higher caries activity in prospective studies of children and adolescents [25].

In the present study, the reported sugar intake was estimated differently due to using dietary data from the food diaries or from the FFQs. Energy percentages calculated from the food diaries indicated that only participants in the high caries group exceeded the recommended sugar intake. When the HDAS was estimated by using FFQ data, low adherence regarding the recommended intake of sugar was shown for all individuals. The different results in sugar intake between the food diaries and FFQ data may be explained by the lack of reported portion sizes in the food diaries. Only a few individuals estimated specific portion sizes, which resulted in standard portions being used in most of the food diaries. Hence, the HDAS calculated from the FFQs may reflect adherence to the recommended sugar intake more accurately for the study group than the reported sugar intake from the food diaries. Thus, since the FFQ captures meal patterns during a longer period, the FFQ results may reflect the intake occasions and food frequencies among these young adults more correctly, which in itself may be an interesting methodological issue.

The study group reported having consumed the recommended amounts of fat, protein, and carbohydrates, but not the recommended amount of fiber. The low fiber intake might be due to the low reported intake of fruit and vegetables, found both in the FFQs and in the food diaries. The low fiber intake may also be due to a low intake of whole-grain products, as reported by the individuals. Associations between a low intake of vegetables and whole meal products and dental caries have been found in previous studies. Sanders et al. [26] found an inverse association between vegetables and whole grains and dental caries in a diverse Lantix adult population. Another study, analyzing data from the National Health and Nutrition Examination Survey 2011–2014, found that participants who met the recommendations regarding intake of fruit, greens and beans and added sugar were less likely to have untreated dental caries [27].

In the present study, most of the individuals had a high BMI corresponding to overweight or obesity. In comparison with Riksmaten adolescents 2016–2017 [28], the present study had a relative frequency of two to three times more overweight and obese individuals. A greater prevalence of overweight and obesity in our group was also seen when compared with a similar age group in the Swedish population, where only 24.4% and 8.9% had been classified as overweight or obese, respectively [28, 29]. Nonetheless, these differences may indicate a possible association between BMI and caries, which has been assessed in previous research. A research group from Saudi Arabia found a positive association between high BMI and high occurrence of dental caries when the patient also had education less than high school, current smoking, or medical issues [30]. Another study found a statistically significant difference between normal-weight caries-free children and pre-obese/obese caries-active children in terms of meal patterns between meals (snacking), where the latter group reported higher frequency of snacking [31]. However, these results are contradicted by two other research groups, which found no statistically significant associations between BMI and dental caries [32, 33]. Further studies are required on the association between BMI and caries and it is necessary to adjust for several confounding factors [34]. Moreover, BMI has been found to be a strong determinant of misreporting in several studies where participants with a higher BMI tend to underreport their energy intake to a greater extent than normal-weight participants [35]. In the present study, the BMI effect on underreporting has not been examined and its impact is therefore unclear.

Most of the study group members reported another ethnicity than Swedish-born and non-highly educated parents. Previous studies have strongly indicated that increased intake and frequency of sugar-rich foods have led to an increased caries risk among children in populations with low socioeconomic status [36, 37]. In a report on social differences in oral health in children and adolescents, the Swedish National Board of Health and Welfare concludes that having immigrant parents from countries outside the Nordic region and Western Europe and parents with primary school only are two of the most important risk factors for dental caries [38]. The present study supports the evidence that children who grow up in less socially favorable circumstances tend to have poorer dental care habits, fewer dental care visits and a poorer diet, which explains the higher prevalence of dental caries [39]. Moreover, this is further emphasized in this study where individuals in the high caries group tend to belong to a poorer socioeconomic position with regard to parental education. Despite the fact that the study setting is in Sweden with its typical sociopolitical structure, the findings of behavior patterns towards dietary intake may be relevant and important for Sweden and other countries as well. However, somewhat contradicting is the fact that the present study participants’ self-reported oral hygiene habits were relatively normal in a population perspective, both regarding tooth brushing/flossing and additional fluoride use.

The present study adds to the existing literature by examining dietary intake and meal patterns among caries-active young adults, an underrepresented group in this field of research. Nevertheless, this study is not without limitations. One limitation is the lack of reported portion sizes, which may have led to an underestimation of the dietary intake. As previously mentioned, the intake of sugar was reported as surprisingly low in relation to the group’s BMI and caries activity. Also, the individuals reported significantly lower energy intake compared with Riksmaten adolescents 2016–2017 [23]. Manually reported portion sizes may not only have affected the estimated sugar intake but also the reported energy intake. Food photos have been judged as appropriate tools to estimate portion sizes at population level [40], and since such tools were not used, the reported energy intake may have been estimated as lower than the actual intake. Gibson et al. [41] have suggested that a weighed food diary is the most reliably validated method to capture actual dietary intake. However, this would have led to a greater burden for the participants and perhaps to more missing data or dropouts. A systematic review evaluating the tendency towards misreported food records found that approximately 15% of the total energy intake was underreported and that there were no differences in misreporting of the energy intake between 24-h recall, estimated food records and weighed food records [35].

One of the main strengths of the present study was that several dietary assessment methods were used. By using two methods, food diary and FFQ, we captured the intake frequency and food amounts at an individual level, by using both prospective and retrospective dietary assessments. Hence, we were able to state that our participants had low adherence to fruit and vegetable recommendations, by using both the food diary and the FFQ. The FFQ has been found to estimate several nutrients with greater accuracy than 24 h recall when validated against biomarkers [42], and the food diary has been estimated to capture the absolute dietary intake better than the FFQ [43]. Another strength was that the FFQ used in this study has been tested for validity [44, 45] and reliability [20]. Lastly, all anthropometrical and clinical measurements were assessed at the dental clinic by a research coordinator who was calibrated by the research group.

## Conclusions

The present study suggests a possible association between snack frequency and dental caries in young adults with high caries activity and showed that most of the individuals in this group has low adherence to healthy dietary guidelines as they report a high intake of sweet food and beverages and an insufficient intake of fruit, vegetables, and dietary fibers. An excessive and high intake frequency of sweet foods is a multidisciplinary issue, and a possible solution may be to lower the recommended intake of sugar further than < 10 E% to prevent dental caries and other diseases among adults [46]. Interdisciplinary treatment collaborations between the dental profession and dietitians could be an alternative to prevent dental caries and other lifestyle-related diseases associated with a high sugar intake and low adherence to dietary recommendations. Follow-up results from the ongoing Dietary and Oral Health study will provide further insights regarding the degree to which individual changes in dietary behavior can be achieved through a dietary intervention provided by a dietitian in a clinical dental setting.

## Data Availability

The data sets generated and/or analysed during the present study are not publicly available due to the Swedish Ethical Review Authority regulations, but are available from the corresponding author on reasonable request.

## Abbreviations

FFQ: The Food Frequency Questionnaire
NNR: Nordic Nutritional Recommendations
HDAS: The Healthy Dietary Adherence score
SFA: Saturated fat
MUFA: Monosaturated fat
PUFA: Polysaturated fat
